# The Use of Single Dose of Rasburicase for the Prophylaxis and Treatment of Tumor Lysis Syndrome in Pediatric Patients: A Narrative Review

**DOI:** 10.3390/hematolrep17060071

**Published:** 2025-12-18

**Authors:** Anselm Chi-wai Lee

**Affiliations:** 1 Paediatric Haematology and Oncology Centre, Hong Kong Sanatorium and Hospital, 2 Village Road, Happy Valley, Hong Kong, China; anselm.cw.lee@gmail.com; Tel.: +852-291-71200; 2 Haematology and Oncology Centre, Department of Paediatrics and Adolescent Medicine, Hong Kong Children’s Hospital, 1 Shing Cheong Rd, Kowloon Bay, Hong Kong, China

**Keywords:** acute lymphoblastic leukemia, Burkitt lymphoma, hyperuricemia, rasburicase, tumor lysis syndrome, urate oxidase

## Abstract

**Background/Objectives**: Rasburicase is licensed for the management of tumor lysis syndrome (TLS) at a daily dose of 0.2 mg/kg intravenously for five days. The use of a single-dose treatment is popular in adult oncology but information in pediatric use is limited. **Methods**: From a literature search, all case reports and series, and comparative studies in which pediatric oncology patients received a single dose of rasburicase were selected for further analysis. Treatment success was determined by normalization of serum uric acid in the absence of serious complications. **Results**: Twelve articles with a total of 243 children were included. A fixed-dose regimen was used in 195, while 153 received weight-based dosing. With fixed dosing, successful treatment was seen in 91.8% and 82.9% at rasburicase doses ≥3 mg and 1.5 mg, respectively (*p* = 0.23). However, there were four mortalities in the lower-dose group. For weight-based dosing, success was observed in 89.2% and 66.7% at doses ≥0.15 mg/kg and <0.15 mg/kg, respectively (*p* = 0.0029). One child required dialysis in the lower-dose group. **Conclusions**: Single dose of rasburicase for the prophylaxis and treatment of TLS in pediatric oncology is an appealing approach with potentially less financial impact and drug toxicity. A fixed dose of at least 3 mg or 0.15 mg/kg by body weight is recommended.

## 1. Introduction

Tumor lysis syndrome (TLS) is a severe metabolic event that complicates rapidly growing and bulky cancers that are often chemosensitive [1,2]. TLS is more commonly seen in hemic malignancies, but is occasionally encountered in patients with solid tumors [3,4]. The rapid cellular turnover and tumor breakdown results in a burst of intracellular electrolytes into the circulation and accelerated purine catabolism. This leads to a decrease in serum calcium while increasing serum potassium, phosphate, xanthines, and uric acid. TLS may occur spontaneously, but more often after the initiation of chemotherapy. Occasionally, a single dose of corticosteroid given as a premedication for treatment or prophylaxis of hypersensitivity reaction prior to blood product transfusion may unexpectedly trigger a full-blown TLS in patients with lymphoid malignancies [5,6]. Acute kidney injury with obstructive uropathy secondary to nephrolithiasis associated with xanthine, urate, or phosphate stones may result in renal shutdown and hypertension. Hyperkalemia may lead to cardiac arrhythmias. Hypocalcemia may result in tetany and seizures. Renal replacement therapy may be indicated for treatment of azotemia, fluid overload, hyperkalemia, hyperphosphatemia, and metabolic acidosis. Mortality may occur as a direct consequence from TLS because of renal or cardiac complications, or multi-organ failure [2].

Hande and Garrow were the first to propose diagnostic criteria for TLS and differentiate the milder, laboratory TLS from the more serious clinical category in 1993 [7]. Patients with laboratory TLS did not generally require additional treatment, whereas those who developed clinical TLS may deteriorate and require dialysis. With these parameters, they were able to show that clinical TLS was uncommon when lymphoma patients were treated with allopurinol. Pre-treatment renal insufficiency and elevated serum lactate dehydrogenase were predictive of the occurrence of clinical TLS. With the availability of new treatments in the new millennium, Cairo and Bishop built on the Hande–Garrow criteria and defined TLS with more relevant parameters [8]. In essence, abnormal biochemistries at baseline, in addition to significant changes from baseline, qualified for the diagnosis of TLS. Also, the time of occurrence spanned for a 10-day period and included spontaneous onset of TLS (Table 1). In the absence of TLS, Cairo and Bishop categorized patients into low-risk and high-risk groups with respect to their propensity to develop TLS according to their underlying malignancy, tumor burden, chemosensitivity, prevailing leukocyte counts, and serum lactate dehydrogenase measurement. Howard et al. further modified the criteria and eliminated the inclusion of fluctuations in biochemistry in the diagnosis. Moreover, at least two biochemical derangements had to be present simultaneously to qualify for the diagnosis of TLS. They also added symptomatic hypocalcemia alone as a parameter for the diagnosis of clinical TLS [9].

Patients who are at risk for TLS are managed with vigorous intravenous hydration therapy and close monitoring of fluid balance and serum electrolyte changes [2,10]. Allopurinol, a xanthine oxidase inhibitor, is recommended to block the production of uric acid. Although uric acid is more soluble at higher urine pH and thus may benefit from urinary alkalinization, the increased risk of precipitation with phosphate stones may offset the potential benefits of adding sodium bicarbonate into the intravenous fluids [2,8]. Despite these measures, severe TLS that necessitates the use of rescue renal replacement therapy still occurs in more than 30% of high-risk non-Hodgkin lymphoma and acute leukemia cases [11]. The introduction of non-recombinant urate oxidase as an extract from Aspergillus flavus started a new chapter of a highly effective treatment for hyperuricemia associated with TLS [12]. By breaking down uric acid into the highly soluble allantoin, urate oxidase was found to be a game-changing compound in pediatric oncology. The non-recombinant product, however, was limited by its supply and potential toxicities related to hypersensitivity [12,13]. In 2002, the recombinant form of urate oxidase or rasburicase was approved by the Food and Drug Administration [13,14] following the successful results published by Goldman et al. [15] and Pui et al. [16] Based on the highly successful results on the management of hyperuricemia, intravenous rasburicase was licenced at a dose of 0.2 mg/kg/day for five days consecutively with the first dose given prior to the commencement of chemotherapy.

Lee et al. were the first group to report on the use of rasburicase as a single-dose regimen in children at high risk for TLS [17]. The cases included two children (aged 11 and 13 years) with acute lymphoblastic leukemia and presenting leukocyte counts >100 × 10^9^/L, and a 4-year-old child with Burkitt leukemia with laboratory TLS. All were treated with a fixed dose of rasburicase 4.5 mg intravenously. The plan was to add on a second dose of rasburicase if the serum uric acid bounced back to abnormal ranges. However, all of them had rapid clearance of uric acid from the blood and the serum levels remained below the lower limit of normal throughout the first week, and hence only a single dose of rasburicase was used in each of them.

In the last two decades, the successful experience of single-dose rasburicase was successfully duplicated in various countries in both adult and pediatric patients with some modifications. In some centers, a fixed single dose was used with doses ranging from 1.5 mg to 7.5 mg [18,19,20]. Others employed a calculated single dose according to the body weight of the recipient [21,22,23]. There was a variation in the dosage that ranged from 0.05 mg/kg to 0.2 mg/kg of body weight. A recent systematic review and meta-analysis found 19 studies on the use of single-dose rasburicase for the management of TLS [24]. The majority of the studies were in adults and only four, including two publications as conference abstracts, were conducted in pediatric patients. Due to the limited number of patients and inaccuracies inherent in this systematic review, no meaningful conclusions were made for the use of single-dose rasburicase in children.

Thus, the purpose of this review is to examine and summarize the use of single-dose rasburicase in pediatric hematology and oncology in the published literature. The review will also analyze if a minimal effective dose can be recommended when single-dose rasburicase is used for the prophylaxis and treatment of TSL.

## 2. Materials and Methods

A literature review was conducted across the databases of MEDLINE, EMBASE, and PUBMED using keyword search with the terms “rasburicase” AND (“single dose” OR “fixed dose”) AND (“child” OR “pediatric”) in October 2025. Additional searches were made by going through the citations from the selected publications. Articles that described or studied the use of single-dose rasburicase for the prophylaxis and treatment of TLS in pediatric oncology patients, 18 years old or less, were included. For studies that included both adult and pediatric patients, only the pediatric data were extracted for the purposes of this study. Conference abstracts were excluded.

TLS was classified as laboratory TLS and clinical TLS as described by the Cairo-Bishop criteria (Table 1), that occurs three days before or seven days after the commencement of anti-cancer therapy [8]. Fixed dosing was defined as the use of the same dose of rasburicase for all patients in the same study irrespective of age or body weight, or a 2-tier system in which two different doses were used according to a pre-defined age or weight. Weight-based dosing was defined as a calculated dose of rasburicase according to body weight, or a two or more-tier system in which progressively higher doses of rasburicase was prescribed in accordance with increasing body weight or age.

Treatment success referred to the normalization of serum uric acid within 48 h of treatment after single-dose rasburicase if the patient was hyperuricemic prior to treatment, maintenance of normal serum levels if the initial uric acid measurement was within normal limits, and without the use of renal replacement therapy, cardiac arrhythmias with interventions, or death of any cause during induction treatment.

The patients’ age, sex, primary diagnosis, occurrence of laboratory or clinical TLS, rasburicase dose and method (weight-based versus fixed), and treatment outcomes were described, as available, from each publication. They were categorized into two groups according to the dosing method of rasburicase, namely, fixed dosing and weight-based dosing. Within each group, patients were further divided into a higher-dose and a lower-dose group. With fixed dosing, the higher-dose group referred to a rasburicase dose of 3 mg or more, while the lower-dose referred to a dose of 1.5 mg or less. For patients treated with weight-based dosing, the higher-dose group corresponded to a dose of 0.15 mg/kg or more, while the lower-dose group corresponded to dose ranges of less than 0.15 mg/kg.

Categoric data were compared using Fisher exact test, 2-tailed, with the GraphPad online calculator (https://www.graphpad.com/quickcalcs/contingency1/. accessed on 19 October 2025). A *p* value of <0.05 was considered significant.

## 3. Results

After deduplication, the literature search yielded 59 publications. Twenty-five publications were excluded as they did not meet the inclusion criteria. Twenty meeting abstracts were also excluded. Of note, 10 of the abstracts were followed by full papers that were included in the later analysis. One case series with combined adult and pediatric patients was excluded because specific outcome data for the pediatric cases were not available. One systematic review and meta-analysis that included both adult and pediatric patients was identified as a reference [24]. The other 12 articles that included a total of 243 pediatric patients treated with single-dose rasburicase for prevention and treatment of TLS were included for analysis.

Among the 12 publications, there were four case reports, with fewer than 10 patients per report, that included 13 children [17,21,25,26], five case series, with 10 or more patients per publication, containing 148 children [27,28,29,30,31], two case series with patients of all ages of which 139 were pediatric patients [32,33], and a comparative study in which 48 children were randomized to receive different doses of rasburicase [34]. The findings were summarized in Table 2.

The exact underlying malignancies were specified in 200 (82.3%) of the 243 children included in this study. The primary diseases included acute lymphoblastic leukemia (97, 48.5%), acute myeloid leukemia (5, 2.5%), unspecified leukemia (34, 17.0%), non-Hodgkin lymphoma (43, 21.5%), and solid tumors (21, 10.5%).

The indications for the use of rasburicase were specified in 209 (86.0%) cases. Treatment for TLS was the indication in 175 (83.7%). Among them, the TLS was classified as laboratory (141, 80.6%), clinical (16, 9.1%), or unspecified (18, 10.3%). The other 34 (16.3%) of the 209 cases were treated with rasburicase when they were considered high risk for TLS.

A fixed dose of rasburicase, ranging from 1.5 mg to 6 mg, was used in 195 cases. At single doses of 3 mg, 4.5 mg, or 6 mg, successful treatment was seen in 45 (91.8%) of 49 cases. The other four patients needed additional doses of rasburicase for the control of hyperuricemia. A dose of rasburicase at <3 mg was used in 146 children, but the response to treatment was only available in 47. Thirty-nine (83.0%) responded successfully to a single dose of rasburicase. The difference was not statistically significant (*p* = 0.23). However, among the 18 children with TLS who received a dose of rasburicase 1.5 mg as reported by Gopakumar et al., two of them required dialysis and succumbed to the complications of TLS [29]. Another two children died from bleeding and infection, respectively. Gopakumar et al. commented that their patients were likely underdosed, noting that 15 of the 18 children received less than 0.15 mg/kg when adjusted by body weight. Philips et al. reported 186 adults and children of which 105 patients were under 18 years of age. All 186 patients received a single dose of rasburicase 1.5 mg. Two patients needed dialysis and there were six mortalities [35]. However, it was not mentioned if any of the pediatric patients were affected by these adverse events.

Weight-based dosing was used in 153 children. The doses included <0.15 mg/kg in 42 (27.5%), 0.15 mg/kg in 17 (11.1%), 0.15–2.0 mg/kg in 27 (17.6%), and 0.2 mg/kg in 67 (43.8%), respectively. Successful treatment was reported in 89.2% (99/111) of the higher dose group who received ≥0.15 mg/kg of rasburicase, whereas 66.7% (28/42) succeeded in the lower dose group (*p* = 0.0029). The results were summarized in Table 3. No serious complications were observed in the higher dose group, but one child who received <0.15 mg/kg of rasburicase needed dialysis [31]. No mortality was reported in either group, however.

As an additional note, Yu et al. performed a systematic review and meta-analysis from 19 clinical studies on the use of single-dose rasburicase in both adult and pediatric patients published before July 2016 [24]. They included 15 adult studies with 906 subjects. Their analysis concluded that a single dose of rasburicase at 6 mg, 7.5 mg, or 0.15 mg/kg consistently and significantly lowered serum uric acid compared with single doses of 4.5 mg or less. The findings were thus supportive of the use of single-dose rasburicase in adult patients. For pediatric patients, only two studies published in a full paper containing 26 children were included. Thus, no specific conclusion could be reached.

## 4. Discussion

Tumor lysis syndrome is a unique combination of metabolic derangements that complicates advanced, rapidly proliferating, and often chemo-sensitive malignancies. The hallmarks of the accelerated cellular breakdown comprise hyperkalemia, hyperphosphatemia, hypocalcemia, and hyperuricemia. Each of these biochemical abnormalities carries its own specific toxicities and together they may jeopardize the cardiovascular system, central and peripheral nervous system, and may cause acute kidney injury. In particular, the precipitation of uric acid crystals and/or calcium phosphate in the renal tubules may lead to acute renal failure and the need for dialysis treatment [1,2,8].

The prevention and control of elevated serum uric acid levels and its crystallization in the renal tubules is the mainstay treatment in TLS in both adult and pediatric patients. The management starts with recognizing TLS as an inherent risk at the time of diagnosis and prior to anti-cancer therapy in most extra-cranial malignancies [2]. The propensity of any patient to develop TLS depends on the underlying oncologic diagnosis and disease burden, the patient’s health status such as age and pre-existing renal disorders, and the planned treatment and its likelihood for rapid response [36]. Hyperhydration (with isotonic fluids >3 L/m^2^/24 h) with forced diuresis to avoid excessive fluid retention and close monitoring and fluid and electrolytes balance is a universally adopted treatment before and during the induction treatment for cancer or leukemia. For patients at low-risk (<1%) for TLS, additional pharmacologic treatment may not be necessary [9,36].

For patients at intermediate- or high-risk (1–5% or >5%, respectively) for TLS, the use of allopurinol, a xanthine oxidase inhibitor that stops the breakdown of hypoxanthine and xanthine into uric acid, is commonly used [36]. Compared with febuxostat, another xanthine oxidase inhibitor, allopurinol is still the preferred treatment because of its lower cost, faster onset of action, and lack of cardiovascular toxicities [2]. Treatment with febuxostat is indicated when the patient is allergic or intolerant to allopurinol, or when the patient is tested positive for HLA-B58:01 that predicts allopurinol hypersensitivity reactions [37]. However, the efficacy of allopurinol in patients at high-risk for TLS is controversial as xanthine is less soluble than urate and hence patients are still at risk for acute kidney injury with xanthine stones [38,39].

The availability of intravenous rasburicase, a recombinant urate oxidase, provides an effective way to treat TLS and to prevent its occurrence in at risk patients. Two landmark studies were published in 2001 that led to its approval by the Food and Drug Administration. Pui et al. [16] treated 131 children and young adults aged 20 years or less with leukemia or lymphoma prospectively. They were given rasuburicase 0.15 mg/kg or 0.2 mg/kg for five to seven consecutive days because of high disease burden or hyperuricemia. A rapid fall in serum uric acid was seen at four hours after treatment and none required dialysis. Goldman et al. [15] randomized 52 children with leukemia or lymphoma, with either large disease burden or hyperuricemia, to treatment with allopurinol or rasburicase. Allopurinol was dosed at 300 mg/m^2^ divided in three daily doses in 25 children and rasburicase was given at 0.2 mg/kg daily for five to seven days in 27 cases. Patients who received rasburicase had significantly lower serum uric acid levels starting from 4 h to 96 h after first dose. Among those with hyperuricemia, the baseline creatinine at 144% of age/sex-defined mean dropped to 102% after 4 days in the rasburicase group. The corresponding measurements were 132% and 147% in the allopurinol, respectively. One patient who received allopurinol treatment required dialysis. As a result, rasburicase treatment was recommended at a dose of 0.2 mg/kg daily for five days for the treatment and prevention of tumor lysis syndrome [13].

A retrospective study reporting on the compassionate use of rasburicase was published in 2001 in which 173 pediatric and 72 adult oncology patients were treated with rasburicase 0.2 mg/kg daily for one to seven days. The data suggested that the treatment might be just as effective when used less than the recommended duration [40]. The successful report by Lee et al., with a single, fixed dose of 4.5 mg prior to chemotherapy, marked the beginning of using single-dose rasburicase in oncology [17]. In the following years, as mentioned in previous sections, alternative regimens using single fixed or weight-based dosing were reported. In 2010, Giraldez and Puto mentioned that most US centers had a protocol of a single, flat dose ranging from 3 mg to 9 mg for the treatment and prevention of TLS [41].

In 2016, Yu et al. [24] published a systematic review and meta-analysis on prospective or retrospective studies and randomized clinical trials of the use of single-dose rasburicase for the management of TLS in either adult or pediatric patients. They identified 15 studies in adult patients. Pooling the data together, their analysis found that, when a single fixed dose of rasburicase was used, the response rates were superior with 6 mg (0.90; 95% confidence interval 0.83–0.97) and 7.5 mg (0.99; 95% confidence interval 0.96–1.00) than lower doses. With weight-based dosing, the response rate from a dose of 0.15 mg/kg (0.94; confidence interval 0.86–1.00) was better than lower-dose regimens.

With respect to pediatric data, Yu et al. [24] drew reference to four publications with a total of 92 children. However, two of the reports were conference abstracts from India in 2013 that were not followed by any full publication [42,43]. Of the remaining two studies, Yu et al. collected 53 cases published by Syrimi et al. [28]. However, a closer look at the publication found that only 19 of them received a single dose of rasburicase. Thus, the analysis concerning pediatric data was inaccurate. Added to the seven cases reported by Latha et al. [26], the systematic review only recovered 26 children managed with single-dose rasburicase and hence no meaningful recommendations could be made.

Nevertheless, with the consistent effectiveness of single-dose rasburicase used at adequate dosages, the regimen is now popular. Compared with the standard dosing, the use of single-dose rasburicase comes with a significant financial advantage. Among adult patients, the adoption of single-dose regimens results in a 90% cost saving or about USD 20,000 per patient prior to the year 2010 [18,22]. Although the absolute saving among pediatric patients is less compared with adults, the percentage and actual cost reduction is still substantial [17,21]. Detailed pharmacoeconomic analysis, however, is beyond the scope of this study.

It should be noted that single-dose rasburicase treatment is not without side effects. In particular, acute hemolysis in subjects with glucose-6-phosphate dehydrogenase (G6PD) deficiency often occurs after the first dose [44,45]. Therefore, the precaution of excluding patients with G6PD deficiency prior to the commencement of rasburicase cannot be over-emphasized [46].

Although the current review is able to gather a total of 243 children treated with a single dose of rasburicase from the medical literature, it is limited by the quality of data due to the heterogeneity of the design, the non-randomized and retrospective observational nature of the studies. As the results in patients given rasburicase prophylactically were not fully reported, it is possible that some of the “at-risk” cases could have been managed with conventional allopurinol and hyperhydration therapy. Further studies including randomized, comparative studies among pediatric patients with predefined risk groups, body weight, number of rescue doses, and costs would be more informative to guide further dosing of rasburicase treatment in childhood cancer management. In this regard, the Howard–Pui definition of TLS [9] is more specific and relevant to real-world practice. Compared with prior defining criteria [7,8], Howard and Pui specified that patients with TLS must meet at least two of the laboratory criteria in the same 24 h period. While prior laboratory definitions would include many situations in which the value would not have any clinical significance in the patients’ status and would not indicate a need to change the patients’ care, Howard and Pui made it mandatory that the defining laboratory results should be abnormal. A mere 25% change in laboratory measurements that did not result in values outside the normal ranges for the patient would not qualify for the diagnosis of TLS.

## 5. Conclusions

Nevertheless, a minimal fixed dose of 3 mg or a calculated dose of 0.15 mg/kg as a single dose appears to be safe and cost-effective in the prevention or treatment of TLS in pediatric hematology and oncology patients.

## Figures and Tables

**Table 1:** Diagnostic criteria for tumor lysis syndrome from the literature.

	Hande–Garrow (1993) [7]	Cairo–Bishop (2004) [8]	Howard–Pui (2011) [9]
Laboratory tumor lysis syndrome	Two of the following within 4 days of treatment:	Two of the following, 3 days before to 7 days after treatment commencement:	Two of the following at the same time, 3 days before to 7 days after treatment commencement:
	Phosphate a 25% increase	Phosphate ≥ 2.1 mmol/L, or 25% increase	Phosphate ≥ 2.1 mmol/L or ULN
	Potassium a 25% increase	Potassium ≥ 6 mmol/L, or 25% increase	Potassium > 6 mmol/L
	Uric acid a 25% increase	Uric acid ≥ 476 µmol/L (8 mg/dL), or 25% increase	Uric acid ≥ 476 µmol/L (8 mg/dL) in adults, or >ULN* in children
	Calcium a 25% decline	Calcium ≤ 1.75 mmol/L, or 25% decline	Calcium, corrected <1.75 mmol/L, ionized < 1.12 mmol/L
	Urea a 25% increase		
Clinical tumor lysis syndrome	Laboratory TLS + one of the following:	Laboratory TLS + one of the following:	Laboratory TLS + one of the following:
	Potassium > 6 mmol/L	Seizure	Seizure
	Creatinine > 221 µmol/L (2.5 mg/dL)	Creatinine ≥ 1.5 ULN*	Creatinine > 1.5 ULN, or ≥26.5μmol/L (0.3 mg/dL) from baseline, or oliguria (<0.5 mL/kg/hour for 6 h)
	Calcium < 1.5 mmol/L		Symptomatic hypocalcemia
	Life-threatening arrhythmia	Cardiac arrhythmia	Cardiac dysrhythmia
	Sudden death	Sudden death	Sudden death probably or definitely caused by hyperkalemia
Remarks	Spontaneous TLS not included	Spontaneous TLS not included	Spontaneous TLS included;
			Abnormalities should not be attributable from other causes

Abbreviations: TLS, tumor lysis syndrome; ULN*, upper limit of normal, per patient age and gender.

**Table 2:** Summary of the 12 case reports and series of pediatric oncology patients treated with single-dose rasburicase.

References	Sex/Age	Diagnosis	TLS	WBC (×10^9^/L)	Urate (mg/dL)	Creatinine (mg/dL)	Rasburicase Dose	Remarks
Case Reports								
Lee [17]	M/11	ALL	Lab	173	13.8	1.3	4.5 mg	Successful
	M/4	BL	Lab	NA	11.9	0.5	4.5 mg	Successful
	M/13	ALL	Lab	198	11.4	?	4.5 mg	Successful
Latha [26]	M/8	ALL	Clinical	5.41	18.4	3	0.15 mg/kg	Successful
	F/7	ALL	Clinical	24.7	30	2.9	0.15 mg/kg	Successful
	M/13	ALL	Clinical	4.67	32.2	2.1	0.15 mg/kg	Successful
	M/13	ALL	Clinical	1.10	23.6	2.6	0.15 mg/kg	Successful
	F/12	ALL	Clinical	0.68	9.7	1.7	0.15 mg/kg	Successful
	M/6	ALL	Clinical	522	31.2	1.8	0.15 mg/kg	Successful
	M/13	ALL	Lab	34.8	10.3	1.4	0.15 mg/kg	Successful
Hooman [25]	M/5	ALL	Clinical	38.7	44	2.8	0.1 mg/kg	Successful
Liu [21]	?/8	ALL	Lab	>400	13.2	0.8	6 mg	Successful
	?/1.5	ALL	Lab	120	8.5	0.5	2.5 mg	Successful
Case series (pediatrics)								
Syrimi [28]	19 cases	ALL/WBC > 100 NHL III/IV NBL HB	Lab 5 HR 14	?	?	?	0.2 mg/kg	Successful prophylaxis after single dose in 15 and >1 doses in 4 when urate rebound > 400 µmol/L
Jayabose [31]	41 cases	ALL 36 NHL 4 AML 1	Lab 36 HR 5	?	>7 in 36	≥1.3 in 9	0.1–0.15 mg/kg	Successful prophylaxis after single dose in 27 and >1 doses in 14 ^#^ 1 needed dialysis
Alavi [30]	48 cases	ALL 22 AML 4 NHL 5 WT 5 Others 12	Lab 45 Clinical 3	?	Elevated in all	Elevated in 3	0.2 mg/kg	Successful after single dose in 44 and >1 doses in 4 ^#^
Appaji [27]	22 cases	ALL 15 NHL 7	Lab 16 Clinical 6	?	10.7–34.5	Elevated in 15	1.5 mg	Successful after single dose in 20 and >1 doses in two ^#^
Gopakumar [29]	18	ALL 12 NHL 6	All had Clinical/ Lab TLS	?	?	?	1.5 mg	6 needed >1 doses; 2 needed dialysis; 2 died from TLS; 1 died from bleeding; 1 died from infection
Case series (pediatric cases extracted from the whole series)								
Gupta [33]	24 (<18 yo) cases (out of 55)	?	? (TLS 12; HR 43)	?	?	?	1.5 mg (≤30 kg) in 6; 3 mg (>30 kg) in 18	Successful after single dose in all
Kukkar [34]	10 pediatric cases (out of 15)	?	HR 10	?	?	?	0.15 mg/kg	Successful after single dose in all (urate < 7.5)
Comparative study								
Savva [35]	48 cases	Leukemia 34 Lymphoma 10 RMS 4	Lab 33 HR 15	?	?	?	WBD: 0.15–0.2 mg/kg vs. FD 6 mg flat	Normalization of urate (<5 mg/dL) at 24 h: WBD, 23/27 (17/21 for patients with TLS) FD, 17/21 (8/12 for patients with TLS) (*p* = 0.715)

Abbreviations: ALL, acute lymphoblastic leukemia; AML, acute myeloid leukemia; BL, Burkitt lymphoma; FD, fixed dosing; HB, hepatoblastoma; HR, high-risk, for evolving into tumor lysis as proposed by Cairo and Bishop [8]; Lab, laboratory; NA, not available; NBL, neuroblastoma; NHL, non-Hodgkin lymphoma; RMS, rhabdomyosarcoma; TLS, tumor lysis syndrome; WBC, white blood cell count; WBD, weight-based dosing; WT, Wilms tumor; yo, years old; ?, not specified. ^#^, The exact number of extra doses of rasburicase was not provided in these reports

**Table 3:** Comparison of the rates of successful treatment after single-dose rasburicase, with as fixed doses or weight-based doses, according to different dose ranges.

	Lower-Dose Group	Higher-Dose Group	*p* Value
**Single, fixed dosing**	**<3 mg**	**≥3 mg**	
Successful/total cases	39/47	45/49	0.23
**Weight-based dosing**	**<0.15 mg/kg**	**≥0.15 mg/kg**	
Successful/total cases	28/42	99/111	0.0029

## Data Availability

The original contributions presented in this study are included in the article. Further inquiries can be directed to the corresponding author.

## Abbreviations

The following abbreviations are used in this manuscript:

ALL	Acute lymphoblastic leukemia
AML	Acute myeloid leukemia
BL	Burkitt lymphoma
NBL	Neuroblastoma
NHL	Non-Hodgkin lymphoma
RMS	Rhabdomyosarcoma
TLS	Tumor lysis syndrome
WT	Wilms tumor
